# Characterization, Thermal Stability and Antimicrobial Evaluation of the Inclusion Complex of *Litsea cubeba* Essential Oil in Large-Ring Cyclodextrins (CD9–CD22)

**DOI:** 10.3390/foods12102035

**Published:** 2023-05-17

**Authors:** Chuan Cao, Peng Xie, Yibin Zhou, Jing Guo

**Affiliations:** 1 Department of Food Inspection and Testing, College of Environment and Life Health, Anhui Vocational and Technical College, Hefei 230011, China; 2 Anhui Engineering Laboratory for Agro-Products Processing, College of Tea and Food Science & Technology, Anhui Agricultural University, Hefei 230036, China; 3 Food Processing Research Institute, Anhui Agricultural University, Hefei 230036, China; 4 Department of Applied Economics, College of Grain and Supplies, Nanjing Finance and Economics, Nanjing 210023, China

**Keywords:** large-ring cyclodextrins, *Litsea cubeba* essential oil, inclusion complex, antimicrobial properties

## Abstract

Food safety issues are becoming increasingly important as a result of contamination with foodborne pathogenic bacteria. Plant essential oil is a safe and non-toxic natural antibacterial agent that can be used to develop antimicrobial active packaging materials. However, most essential oils are volatile and require protection. In the present study, LCEO and LRCD were microencapsulated through coprecipitation. The complex was investigated using GC-MS, TGA, and FT-IR spectroscopy. According to the experimental results, it was found that LCEO entered the inner cavity of the LRCD molecule and formed a complex with LRCD. LCEO had a significant and broad-spectrum antimicrobial effect against all five microorganisms tested. At 50 °C, the microbial diameter of the essential oil and its microcapsules showed the least change, indicating that this essential oil has high antimicrobial activity. In research on microcapsule release, LRCD has proven to be a perfect wall material for controlling the delayed release of essential oil and extending the duration of antimicrobial activity. LRCD effectively extends antimicrobial duration by encasing LCEO, thus improving its heat stability and antimicrobial activity. The results presented here indicate that LCEO/LRCD microcapsules can be further utilized in the food packaging industry.

## 1. Introduction

Essential oils are aromatic oily liquids that are extracted from aromatic plants by means of distillation, extrusion, and solvent extraction. Alkaloids, flavonoids, monoterpenes, sesquiterpenes, and other naturally occurring compounds are abundant in plant essential oils. They have effective broad-spectrum bactericidal characteristics, and it is difficult for essential oils to render people resistant to drugs. Their antimicrobial properties make them important ingredients for the cosmetic, healthcare, agricultural, and food industries [1]. However, because they differ in their chemical makeups and contents, many essential oils have different types of functions. The evergreen shrub *Litsea cubeba* (*Lour.*) *Pers.* is indigenous to southern China, Japan, and Southeast Asia. As one of the most important economic spice plants in China [2], its fruit can be used as a herbal medicine to alleviate inflammation, respiratory conditions, and gastrointestinal discomfort [3]. *Litsea cubeba* essential oil (LCEO) is a light-yellow liquid with a strong lemon flavor [4] and a pungent aroma. LCEO has been shown to inhibit the development and reproduction of *Saccharomyces cerevisiae*, an organism that causes food spoilage, as well as the generation of aflatoxin [5]. Additionally, LCEO and its derivatives have bacteriostatic effects against plant pathogens such as anthracnose, watermelon fusarium wilt, and rice blast; the higher the concentration is, the greater the effect is [2]. Tiwari [6] tested LCEO as a food preservation agent and found that it had an antimicrobial effect against foodborne microbes. Moreover, LCEO may be utilized as a flavoring agent for cosmetics or food products [1], and it can also be used to extract citral, vitamins A, E, K, and ionone [7,8]. However, due to their low water solubility, high volatility, and sensitivity to heat and oxygen, essential oils are not commonly used as food preservatives. To expand the application scope of essential oils in the food, medicinal, and cosmetic sectors, it is critical to investigate the microencapsulated inclusion compounds of essential oils. The purpose of encapsulation is to protect the core materials or their properties from external influences (such as light, heat, oxygen), improve the stability of essential oils, control their release, and extend their shelf lives by combining active ingredients with wall materials [9,10].

It has been reported that microcapsules have been used to continuously release essential oils over the past few years. Due to their non-toxic and controllable characteristics, cyclodextrins have gained a great deal of attention among other embedding methods [11]. In comparison with small-ring cyclodextrin, large-ring cyclodextrin (LRCD) has a larger hydrophobic cavity, a unique flexible form, and superior water solubility [12]. It can increase accessibility to the molecular inclusion process. For instance, some drugs or potent guests may form complexes poorly with CD_6_–CD_8_ but well with CD_9_–CD_12_ [13]. The improved stability or solubility of these guest molecules can be explained by the relationship between the guest and LRCD molecules during the embedding process. The guest molecules are momentarily trapped or encased in the host cavity to produce modifications beneficial to the visitor molecules. In recent years, researchers have observed that LRCD may combine with the acetate of vitamin E to generate a compound that increases its solubility [14]. However, a limited number of publications are available on the use of LRCD in complex essential oils and the assessment of inclusion compounds.

The objective of this study was to encapsulate LCEO with LRCD to evaluate the complex’s thermal stability in food and medicine. Additionally, LCEO was assessed for its antimicrobial activity, as well as its stability and inhibitory activity on the investigated strain via direct contact and fumigation. The kinetics of the release of essential oils from the microencapsulation was investigated by fitting a kinetic equation to a volatilization curve.

## 2. Materials and Methods

### 2.1. Materials

The laboratory synthesized LRCD (9–22) and determined it using MALDI-TOF-MS (New ultrafle Xtreme, Bruker Daltonics Inc., Billerica, Germany). *Litsea cubeba* essential oil was purchased from Shanghai Yuanye Biotechnology Co., Ltd., Shanghai, China. CAS No. 68855-99-2, MDL No. MFCD 00678555. The test strains were obtained from Shanghai Conservation Biotechnology Center. The concentration of *Escherichia coli* (ATCC 8739), *Bacillus subtilis* (ATCC 6633), and *Kluyveromyces marxianus* (ATCC 36534) was 10^6^–10^7^ CFU/mL. The spore suspension of *Aspergillus niger* (ATCC 16404) and *Rhizopus oryzae* (ATCC 6227b) with the fungal concentration of 10^4^–10^5^/mL was obtained by counting the blood cells, while the other chemicals and reagents were obtained from Sinopharm Chemical Reagent Co., Ltd. Beijing, China.

### 2.2. LCEO/LRCD Inclusion Complex Preparation

The preparation of the inclusion complex was modified according to the previous method [15]. LRCD (2 g) was completely dissolved in distilled water (10 mL) and stirred at 55 °C for 30 min using a collecting magnetic stirrer (DF101S; Gongyi Kerui Instrument Co., Ltd., Gongyi, China). Then, we added 1 mL LCEO (dissolved in 2 mL ethanol) to the cooled LRCD solution. Subsequently, we vortexed the combined solution in an eddy current oscillator for 5 min and kept it at 4 °C for 12 h. Vacuum freeze drying (LC-10N-50B Shanghai Lichen Instrument Technology Co., Ltd., Shanghai, China) was used to recover the precipitated LCEO/LRCD inclusion compound. An established procedure was used to determine the amount of LCEO included in the inclusion compound [12]. To dissolve 100 milligrams of the substance, 5 mL of absolute ethanol was employed. Anhydrous sodium sulfate was subsequently added, and then the mixture was diluted with 10 mL of absolute ethanol. The combination was then left to stand for 24 h in order to give the active ingredient time to dissolve in the solution. Prior to measurement, all traces of LRCD were eliminated from the solution, leaving the active ingredient alone. UV spectrophotometry was used to determine the total essential oil content by measuring the absorbance at 244 nm.

The formula below was used to determine the EE parameters:

$$\mathrm{EE}=\frac{Amount\;of\;entrapped\;active\;compound}{initial\;amount\;of\;essential\;oil}\times 100$$

At a molar ratio of LRCD/CEO of 6, the inclusion compound had the maximum inclusion efficiency, reaching 85.83%.

### 2.3. Structure and Physicochemical Properties of LCEO/LRCD

#### 2.3.1. Determination of the FTIR of LCEO/LRCD

The samples were analyzed using FT-IR spectroscopy (IS 50, Thermo Nicolet Corporation, Madison, WI, USA). The materials were combined with dried KBr in particular proportions (1:100, *v*/*v*) to create tablets [16]. The scanning range was 400–4000 cm^−1^, and the resolution was 4 cm^−1^.

#### 2.3.2. Thermodynamic Determination of LCEO/LRCD

A total of 5 g of sample was weighed, and we performed thermogravimetric analysis using a TGA Q600 (TA Instruments, New Castle, DE, USA), followed by scanning from 25 to 700 °C at a heating rate of 10 °C/min and a nitrogen flow rate of 50 mL/min. The test was performed using a NetzschSTA-449C instrument under the following conditions: a nitrogen atmosphere, temperature range of 25–500 °C, and temperature programmed rate of 15 °C/min [12].

#### 2.3.3. Observation of the LCEO/LRCD Microstructure

Hitachi field emission SEM was used to investigate the particle shape of the LRCD-encapsulated aromatic compounds (SEM S-4800, Hitachi, Tokyo, Japan). We examined the sample particles’ micromorphology and structure. The sample must be sprayed with a 1.0 kV accelerating voltage and magnified 1000 times [17].

#### 2.3.4. Analysis of the Volatile Profile

We weighed 5 g of essential oil and microcapsules containing the same amount of essential oil into a headspace extraction bottle, placed it in a thermo-static oscillator with a 150 g oscillation frequency, a used solid-phase microextraction fiber (Supelco, Bellefonte, PA, USA) to perform extraction at 60 °C for 40 min, collected the volatile compounds in the LCEO, and analyzed them with GC-MS (QP2010 Shimadzu, Kyoto, Japan) and a DB-5ms capillary column (Agilent Technologies Inc., Palo Alto, CA, USA).The carrier gas was nitrogen, and the flow rate was 1 mL/min. The temperature increase program moved from 40 °C to 180 °C at 5 °C/min, the temperature was maintained at 10 °C/min to 230 °C for 5 min, and the temperature at the injection port was 230 °C. The compounds were identified in the preliminary stage by comparing the mass spectra to real samples from the NIST MS collection [18]. Normalized quantification was carried out with the peak area of the chromatographic peak of the total ion flow of GC-MS, and the identified components accounted for 99.42% of the total area of the chromatographic peak.

### 2.4. Inhibitory Activity of LCEO and Its Microcapsules toward the Test Strains

#### 2.4.1. Determination of the Diameter of the LCEO Direct Contact Inhibition Strain

A nutrient agar (NA) medium slant was used to inoculate E. coli and Bacillus subtilis, which were then activated at 37 °C for 24 h before being cultivated in LB (Luria Bertani) liquid medium for 24 h at 37 °C [19]. Finally, a bacterial suspension with a bacterial concentration of 10^6^ to 10^7^ CFU/mL was obtained using 10-times gradient dilution as a standby.

We scraped a small amount of yeast onto the slope of the MEA solid culture (Malt Extract Agar), where it was left to be activated for 48 h. The activated yeast was then cultured in MEB (Malt Extract Broth) liquid medium for 48 h at 28 °C. Finally, a yeast suspension with a concentration of 10^6^–10^7^ CFU/mL was obtained after performing a 10-fold gradient dilution as a standby.

We scraped a small number of Aspergillus niger and Rhizopus oryzae spores onto an inoculation ring, inoculated them onto the slope of the PDA medium (Potato Dextrose Agar), and activated them at 28 °C for seven days. Then, using an inoculation ring to scrape out the spores, we added a sufficient amount of sterile physiological saline to the inclined surface and counted the spore suspension using a blood cell plate to acquire a concentration of 10^4^–10^5^ pieces/mL as a standby.

(1) LCEO direct contact inhibition strain test

Using a pipette gun, 100 μL of suspension was added to a sterile and freshly solidified LB (culture *E. coli* and *Bacillus subtilis*)/MEA (culture yeast)/PDA (culture Aspergillus niger and Rhizopus oryzae) plate, covered uniformly with a sterile coating rod, and allowed to dry before a 6 mm sterile filter paper sheet was added to the plate’s center. We diluted the natural essential oil or microcapsule to a concentration of 20 mg/mL with 1% Tween-80 solution and transferred. Then, 20 μL diluent was placed on a filter paper sheet, and an equal amount of sterile water culture medium was used as the control group. The diameter of the inhibitory microbiota was measured using the cross method. *Bacillus subtilis* and *Escherichia coli* were grown inverted at 37 °C for 24 h, whereas *Rhizopus oryzae*, *Aspergillus niger*, and *yeast* were grown at 28 °C for 2–7 days [19].

(2) LCEO fumigation inhibition strain experiment

A 6 mm sterile filter paper sheet was then placed on the cover of the culture dish after 100 μL of suspension was spread equally onto a sterile and solidified fresh LB/MEA/PDA plate and dried [20]. The natural essential oil or microcapsule was diluted to a 20 mg/mL concentration with 1% Tween-80 solution and transferred. Then, 20 μL diluent was placed on a filter paper sheet, and the culture dish was sealed with a sealing film. As a control, sterile water was added to the culture medium in equal proportions. We measured the diameter of the inhibitory microbiota using the cross method. *Yeast*, *Aspergillus niger*, and *Rhizopus oryzae* were cultured at 28 °C for 2–7 days, whereas *Escherichia coli* and *Bacillus subtilis* were grown upside down at 37 °C for 24 h.

#### 2.4.2. The Inhibitory Activity of Essential Oils and Microcapsules towards Microbes Is Affected by Temperature

The technology of filter paper diffusion was used. The LCEO and inclusion compound were packed and exposed to temperatures of 4 °C, 50 °C, and 100 °C for 1 h before being cooled to ambient temperature. The control group received no treatment.

#### 2.4.3. Determination of the Activity of LCEO and Microcapsule in the Direct Contact Inhibition of the Strains

MIC (minimum inhibitory concentration): The twofold dilution method was used to calculate the microcapsule’s MIC value against five distinct types of microorganisms. We added 0.5 mL culture medium and microbial liquid (100 μL) and 0.5 mL microcapsule diluent (starting concentration: 20 mg/mL) to a 1.5 mL LEP tube, set the temperature at 37 °C (culture *Escherichia coli* and *Bacillus subtilis*) or 28 °C (culture *Yeast*, *Aspergillus niger*, and *Rhizopus oryzae*), and incubated the tube for 72 h in a constant-temperature shaker at 200 r/min. The MIC value was calculated using the lowest concentration that showed no signs of turbidity or fungus formation. The positive control used sterile water in place of the microbiological fluid, whereas the negative control used microcapsule wall material in place of the microscopic capsules.

Minimum bactericidal concentration (MBC) and minimum fungicidal concentration (MFC): Using the viable count technique, the MBC or MFC values of each microcapsule against the five test strains were determined. In the MIC experiment, we chose the group that showed no mycelial growth at all and counted the microbes. The MBC/MFC value represents the lowest concentration of undiscovered colonies [20].

### 2.5. Research on the Kinetics of LCEO and Microcapsule Release

Several adjustments were made to Mehdi and Stefani’s methodology for determining the release kinetics of essential oils and their microcapsules [19,21]. We weighed 1.5 g of microcapsule and placed it in a conical flask with 120 mL phosphate-buffered solution (PBS)–ethanol (3:2 *v*/*v*) solution of pH 6.5 and slowly stirred it with a collecting magnetic stirrer at constant temperatures (37 °C and 65 °C). We extracted 4 mL of sample every 12 h between 0 and 144 h for analysis, replacing it immediately with an equivalent volume of fresh media. Then, we centrifuged it at 5000 rpm for 10 min, collected the supernatant, measured the absorbance value of LCEO at 244 nm, and used the PBS–ethanol (3:2 *v*/*v*) solution as the blank control. The release rate Q of the essential oils and microcapsules was obtained using the following formula [22]:

(1) $$Q=\sum_{t=0}^{t}\frac{Mt}{M0}\times 100$$

where *Mt* is the cumulative amount of essential oil released at each sampling time.

*M*0 is the initial mass of the essential oils in the sample.

### 2.6. Evaluation of LCEO Antimicrobial Activity and Microcapsule Sustained Release

#### 2.6.1. Release Study

In order to explore the release mechanism and kinetics of the essential oil microcapsules, the zero-order kinetic model, first-order kinetic model, Higuchi equation, and Peppas equation were used, according to the research of Dima, C. and Bezerra, F.M. [22,23]. Four model techniques were investigated to characterize the release kinetics of the essential oils and microcapsules, including the zero-order, first-order, Higuchi, and Peppas empirical models. Origin 2018 software (OriginLab Corporation, Northampton, MA, USA) was used to fit the release kinetic equation.

#### 2.6.2. Antimicrobial Activity

The antimicrobial activity of the LCEO and microcapsules was modified according to previously described methods [24]. Taking *Escherichia coli*, *Aspergillus niger*, and *Rhizopus oryzae* as the test strains, the exposure timed were determined to be 0 d, 7 d, and 14 d, respectively, according to the MIC determination results and the volatile curve of the essential oil microcapsules. The experiment was carried out in triplicate on a filter paper diffusion culture plate (90 mm in diameter). Fresh blocks of *Aspergillus niger*, *Rhizopus oryzae*, and *Escherichia coli* were inserted in the center of the sterile, coagulated LB (culture *Escherichia coli*)/PDA (culture *Aspergillus niger*, *Rhizopus oryzae*) media. After that, the culture was inverted onto the top of the culture dish containing the essential oil or microcapsules. A sealing film was used to cover the culture dish. Using the cross technique, the culture was inverted at 37 °C or 27 °C for 0, 7, and 14 days to determine the diameter of the microbial circle. The blank control group received neither essential oil nor microcapsules.

### 2.7. Statistics and Data Analysis

The results were measured in triplicate and expressed as the mean ± standard deviation (SD). Duncan’s multi-range test was performed to examine the significant differences between the mean values using the statistical program SPSS version 21.0 (SPSS Corporation in Chicago, IL, USA).

## 3. Results and Discussion

### 3.1. Analysis of LCEO Microcapsules

#### 3.1.1. Analysis of Fourier Infrared Spectra

**Figure 1:**
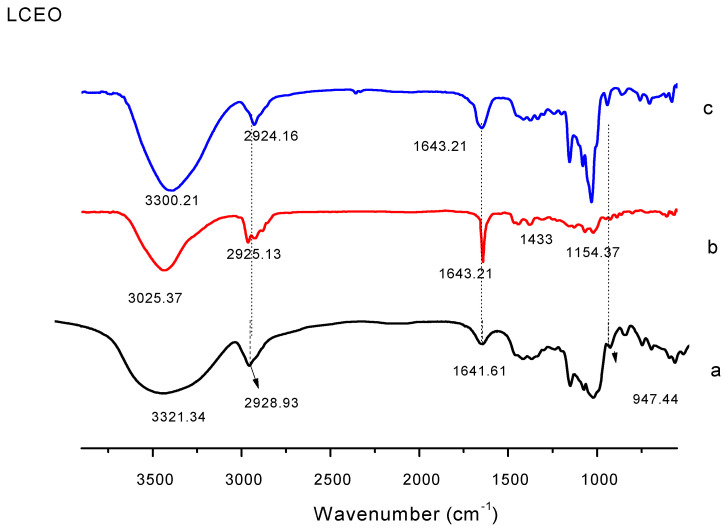
FTIR spectra of LRCD, LCEO, and inclusion complex. (**a**) LRCD, (**b**) LCEO, (**c**) inclusion complex.

FT-IR spectroscopy is usually used to confirm the presence and interaction of guest molecules in the wall material [25]. The infrared spectra of the LCEO and microcapsules are shown in Figure 1. There are numerous, clearly distinguishable absorption peaks in the LRCD infrared spectrum (a). The peak at 3321 cm^−1^ is attributed to the tensile vibration of the OH group, the characteristic spectral band at 2928 cm^−1^ corresponds to the tensile vibration of the CH_3_ group, and the peak at 1641 cm^−1^ is a reaction to the bending vibration of H-OH. In the infrared spectrum of LCEO (b), the characteristic peak at 2925 cm^−1^ is attributed to the vibration of CH_2_ [2], and the typical strong absorption band at 1643 cm^−1^ is related to the tensile vibration of the C=O group in the citral compound. This prominent peak indicates that LCEO is contained in the cavity of LRCD. According to the characterization of the infrared spectrum, there is a strong interaction between cyclodextrin and guest molecules. Here, the significant changes in the characteristic bands of pure matter demonstrate that LCEO entered the inner cavity of the LRCD molecule and formed a complex with LRCD.

#### 3.1.2. Observation of Appearance and Structure

**Figure 2:**
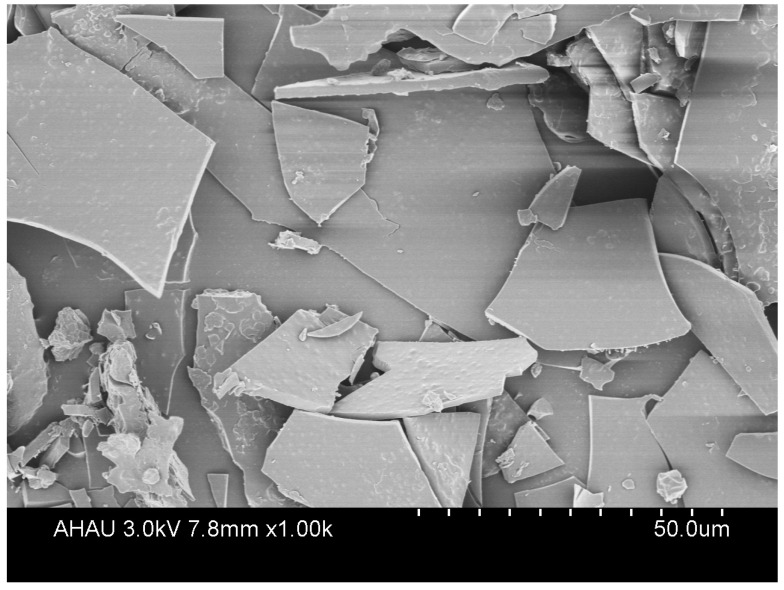

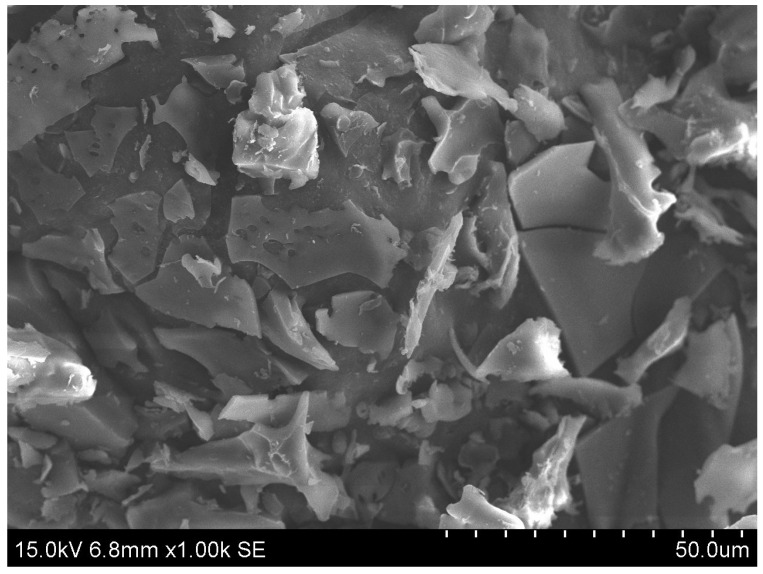
SEM of LRCD and inclusion complex.

Figure 2 shows the particle morphology of LRCD and LCEO/LRCD. It is possible that LRCD could serve as a suitable wall material for the formation of inclusion compounds by guest compounds [26]. In its pure form, LRCD appears as an irregular sheet. Compared to LRCD, the particle size and shape of the embedded material changed, indicating the presence of guest molecules and mixed fragments, which may indicate the formation of a complex [27]. Kringel [28] demonstrated that the particle size and shape of the inclusion compound were altered as a result of combining the wall and core materials. Therefore, the morphology of the inclusion complex exhibits aggregated particle clusters due to the hydrophilic nature of LRCD and its V-shaped structure, which might prevent guest molecule adhesion and effectively encapsulate guest molecules.

#### 3.1.3. Thermogravimetric Analysis

**Figure 3:**
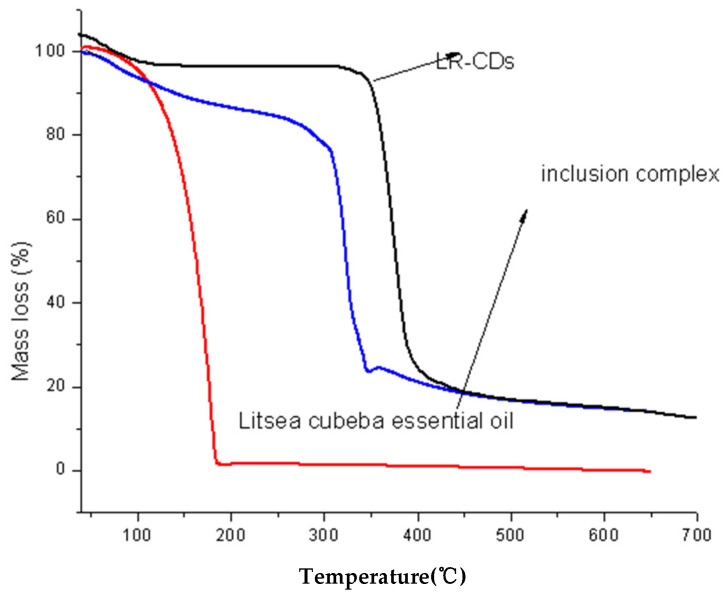
Thermogravimetric analysis diagram of the LRCD, LCEO, and inclusion complex.

TGA is a method used to assess the thermal stability of various compounds following thermal degradation [29]. Figure 3 depicts the thermogravimetric analysis of the LCEO and microcapsules. According to the thermogravimetric investigation, the mass loss of LRCD occurs in two stages. The first stage involves moisture loss (7.27 percent loss) at temperatures between 40 and 100 °C. The second stage might be attributed to the degradation of LRCD from 280 to 360 °C (loss 74.66%), as previously reported [30]. LRCD wraps guest molecules efficiently and stably, even at high temperatures, enhancing thermal stability and limiting essential oil evaporation [31]. The weight of LCEO decreases by 98.63% as the temperature approaches 192 °C. For the LRCD/LCEO complex, the TG curve is similar to that of the LRCD, but the distributions interval of thermal degradation are slightly different; the distributions of the first and second weight loss are in the ranges of 40–93 °C and 292–343 °C, respectively. Consequently, the LCEO’s thermal stability is increased when it forms a complex with the LRCD.

#### 3.1.4. Analysis of Volatile Compounds

GC-MS is applied to detect volatile components in essential oils or products. Wang prepared a *Litsea cubeba* essential oil and β-cyclodextrin inclusion complex and investigated its chemical composition using GC-MS. A total of 20 chemical constituents were identified, accounting for approximately 96.36% of the overall composition of essential oils [2]. In our study, we prepared LCEO microcapsules using LRCD and compared the main volatile compounds of the LCEO and inclusion compounds, and the results are shown in Table 1. Both the inclusion complex and the original LCEO included 29 compounds and 22 components, respectively, with higher relative contents. Because LCEO has different encapsulation capabilities in the LRCD cavity, the amount of LCEO components in the microcapsules was lower than that in the free LCEO, a finding which is consistent with a previous report [18] and suggests that a small amount of essential oil was lost during the encapsulation process. According to the results of the composition analysis, the principal constituents of LCEO are monoterpene compounds and their oxides, including α-Citral (20.17%), β-Citral (17.08%), limonene (15.94%), β-pinene (5.83%), and β-hyacinene (4.34%), with a total mass fraction of 63.36%. A few minor changes occurred in the proportions of major components in the microcapsule; limonene (21.56%) has the largest percentage, followed by α-Citral (20.09%) and β-Citral (16.91%). The evaporation and degradation of substances may have contributed to this phenomenon, or the peak areas of some compounds may have decreased, thereby resulting in the rise in the peak areas of other compounds [32]. In this study, variations in the content of linked components within the core material may have been caused and/or affected by the mixture of wall and core materials, although the fundamental components were still discernible.

### 3.2. Analysis of the Inhibitory Effects of LCEO and Its Microcapsules on the Strains

#### 3.2.1. Analysis of the Diameter of the Inhibitory Region of LCEO and the Effect on the Strain

LCEO inhibits the growth of the five selected strains of microorganisms, as shown in Table 2. The results indicate that the inhibition zone of the strains has a diameter of 12.8–23.2 mm. In terms of the size of the inhibition zone, the inhibition effect on mold (*Aspergillus niger*) is better (diameter > 20 mm). The inhibition effect on *Kluyveromyces marxianus* is the worst, and the inhibition effect is consistent with that established through direct contact. One possible reason for this is that the main constituent of LCEO essential oil is citral, which has a strong inhibitory effect on microorganisms by destroying their membranes and cell walls. Various essential oils have distinct inhibitory properties, and the composition and amount of essential oil impact the inhibition properties.

#### 3.2.2. The Inhibition Effect of Essential Oils and Microcapsules on the Test Strain Is Affected by Temperature

Table 3 shows the diameter of the inhibition zone of the strain after one hour at 4 °C (low-temperature environment), 50 °C (high-temperature environment), and 100 °C (food-processing environment). The essential oils and their microcapsules exhibit the least change in their inhibition zone diameter at 50 °C. The five tested strains were inhibited significantly by the essential oils as well as their microcapsules when treated at 4 °C and 100 °C, whereas the microcapsules had a lesser effect and exhibited better stability than the essential oils. As a result of the different temperature treatments, LCEO had the greatest effect on *Bacillus subtilis bacteriostasis*, reducing the maximum diameter by 2.38 mm, while it did not have any effect on *Aspergillus niger* or *Rhizopus oryzae*. At different temperatures, the inhibition effect of the strain may differ due to differences in the volatility and stability of the antimicrobial active components of the essential oils. Volatility is affected by low temperatures, but the disintegration of volatile components is caused by temperatures over 100 °C.

#### 3.2.3. Analysis of the Activity Inhibition of LCEO and Its Microcapsules in Direct Contact with Strains

The MIC and MBC are indicators of the resistance of strains. The lower the number, the stronger the resistance of the substance to the test strain. Table 4 presents the results of the determination of MIC and MBC for the LCEO and microcapsules. The LCEO and microcapsules have good resistance to the five tested strains. The MIC value of the LCEO/LRCD microcapsules for *yeast* and *Aspergillus niger* is the same as the MBC value, being 7.0 mg/mL and 3.6 mg/mL, respectively. However, the LCEO microcapsules have high MBC values for *Escherichia coli* and *Bacillus subtilis.* This difference can be explained by the different resistance and sensitivity of the microbes to LCEO. As a result, LCEO has a greater MBC than MIC.

### 3.3. Essential Oils’ Microencapsulation Release Performance

#### 3.3.1. Determination of the Essential Oil Microcapsule Volatile Curve

**Figure 4:**
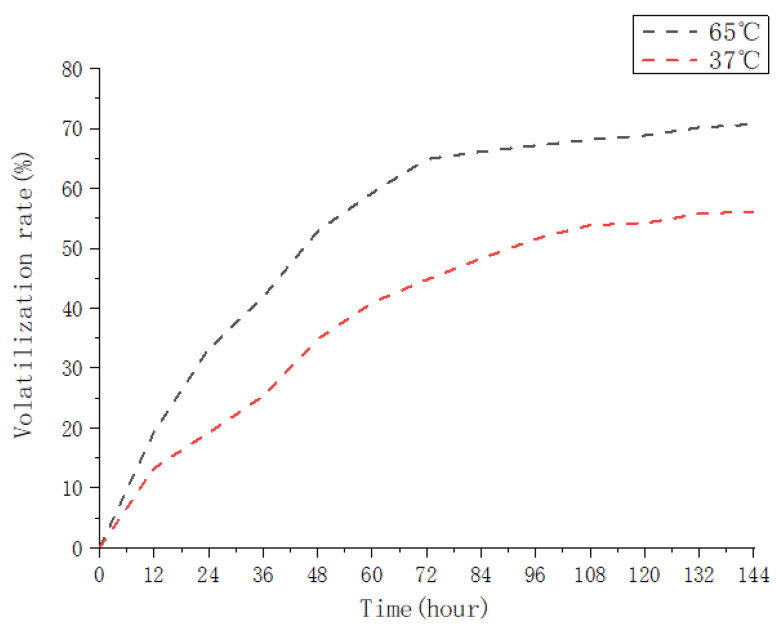
Volatilization curves of the inclusion complex at 37 and 65 °C.

The temperature of the environment had a significant impact on the sustained release performance of the essential oil microcapsules. Figure 4 depicts the volatilization curves of the essential oil microcapsules at 37 °C and 65 °C. It is evident that the release cycle of the essential oil microcapsule is divided into two stages. The first stage corresponds to LCEO encapsulation of more than 60% released at 65 °C and more than 40% released at 37 °C when the fast-release stage reaches 72 h, and the release rate gradually increases to a constant value after 144 h. The “explosive impact” of fast microcapsule release is demonstrated. A significant amount of oil is released in a very short period of time when the oil is in contact with water [33]. The burst effect results in a significant volume of oil being released as a result of the oil molecules’ rapidly disintegration on the surface of the microcapsule or the biopolymer. As a result of their microscopic particle size and thin biopolymer matrix, the microcapsules burst. In the presence of a solution in the environment, water will be able to permeate the microcapsule wall, resulting in a conversion of the polymer from glass to rubber [34].

It appears that the essential oils’ release rate slows in the second stage or reaches a steady state. It is possible that the microcapsule cavity prevents the release of essential oils. The essential oils are released primarily through diffusion into the polymer matrix, resulting in the further expansion and degradation of the wall material. As a result, the essential oils are contained within a microcapsule in the wall material’s hollow, minimizing their contact with the exterior environment. It is thus demonstrated that LRCD is an effective wall material for controlling the delayed release of essential oils.

#### 3.3.2. Release Kinetics of Essential Oils Microcapsules

Table 5 displays the release kinetics of the essential oil microcapsules at two temperatures, 37 °C (human body temperature) and 65 °C (food processing temperature). The essential oils’ release rate at 65 °C is higher, and the release rate curve of the essential oils at different temperatures most closely resembles the Peppas equation (R^2^ = 0.95). Temperature variations alter the rate of essential oil release without influencing the mechanism of release. Based on the diffusion index “n” of the microcapsules, Peppas’ equation may be used to classify the release mechanism as follows: *n* = 0.85 zero-order release kinetics (case II transport); *n* = 0.43 Fickian diffusion (case I transportation); 0.43 < n < 0.85 abnormal or non-Fickian transport [22,35]; and “supraII” type migration, which occurs when n > 0.89 [36]. It can be seen that at 65 °C, the microcapsule system *n* = 0.43 exhibits Fickian diffusion. There is no linear relationship between the diffusion temperature and the concentration of wall material. The release rate is mostly determined by diffusion, which can be caused by inadequate swelling and the presence of oil droplets on the microcapsule’s surface or outer layer. The microcapsules feature a non-Fickian transport mechanism at 37 °C, and the release rate is determined by the non-Fickian expansion and diffusion process. Diffusion swelling governs the rate of release. As a result of the swelling of the biopolymer matrix and the diffusion of the oil throughout the matrix, essential oils are released [33]. The release rate of the essential oil in the microcapsule changes as the temperature changes. Meanwhile, the Kinetic Equation of release differs. The release rate is related to temperature. The release time of the essential oil in the microcapsule is strongly connected to the release time. Based on the Hippas equation and the first-order kinetic equation, the volatilization curve was fitted. Simultaneously, the greater the temperature is during the release process, and the closer to the first-order kinetic equation and lower the temperature are, the more consistent this process is with the Peppas equation.

### 3.4. Sustained Release Impact on the Antimicrobial Activity of LCEO and Its Microcapsules

**Figure 5:**
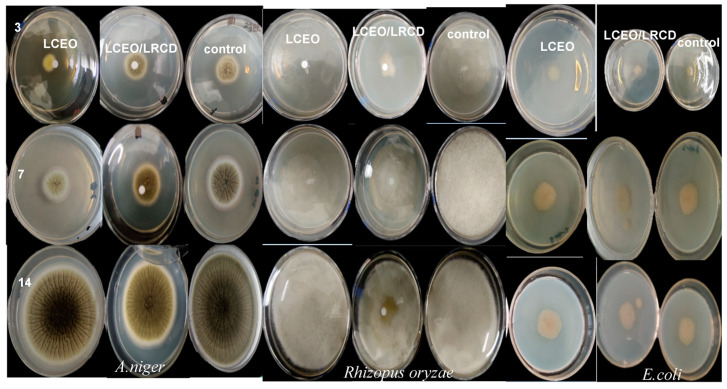
Effects of LCEO and the inclusion complex on the growth of three tested microorganisms with different sustained release times.

Figure 5 and Table 6 show the sizes of the microbial circles of the tested strains inhibited by the essential oils and microcapsules in different sustained release periods. The data in Table 6 indicate that, in various slow-release time periods, the diameter of the bacteria or fungi in the experimental group was significantly lower than that in the blank control group, demonstrating that the essential oil and microcapsules were capable of effectively inhibiting the growth of the bacteria or fungi. Furthermore, after 3 days of incubation, the essential oil treatment group’s bacterial or fungal rings were noticeably larger than those in the microcapsule treatment group, and the essential oil’s inhibitory efficacy was superior to that of the microcapsules. The microcapsules’ superior inhibitory action became apparent when the culture time was extended. The tested strain in the microcapsule group had a lower diameter after 14 days of culture than the essential oil group, particularly in the *E. coli* group, and its bacteriostatic diameter was only 31.74 mm. This distinction demonstrates that microcapsules have a more potent and persistent release impact than pure essential oil in terms of their antimicrobial effectiveness. The main reason for this may be that the CD wall material can effectively reduce the rate of volatilization of the core material and may continually release a specific quantity of essential oil, which inhibits the development of the test strain to varying degrees.

In this study, cyclodextrin was observed to be an appropriate wall material for embedding flavor components; however, small cyclodextrins are rigid due to their lack of pores and place restrictions on the embedding of flavor components. The chemical characteristics of the visitor molecules will be quickly sealed in the cavity of the LRCD when it is utilized as a wall material, resulting in favorable alteration, enhancing stability, and controlling the release of essential oils. As a result, the creation of LRCD has become an emerging topic of research. For antimicrobial applications, microcapsules produced from a combination of LRCD and *Litsea cubeba* essential oil were utilized. An analysis was performed on the structure, properties, and antimicrobial activity of the microcapsules. As a result of the enhancement of the essential oil’s antimicrobial properties after microencapsulation, the use of LRCD as a wall material has some theoretical and practical support.

## 4. Conclusions

The current work indicated that LCEO and LRCD were successfully microencapsulated using coprecipitation for the first time. The complex was analyzed using FT-IR, TGA, and GC-MS. Simultaneously, the influences of essential oil and microencapsulation on the inhibitory performance of five microorganisms were investigated, and the release kinetics after microencapsulation was explored. The analyses indicated that the particle size and shape of the embedded material changed as compared to LRCD, indicating a combination of guest molecules and mixed fragments, and the weight loss rate of the LCEO/LRCD complex was better and more ideal than that of the LCEO. The essential oil and microcapsules of *Litsea cubeba* were found to have 29 compounds and 22 components, with higher contents according to the GC-MS component analysis, these mainly being monoterpenoids. α-Citral, β-Citral, limonene, β-pinene, β-hyacinene were the chemicals with the highest concentration of monoterpenoids. It can be inferred that LCEO enters the inner cavity of the LRCD molecule and forms a complex with LRCD. A potent and broad-spectrum inhibitory effect on the five strains tested was found for LCEO and its microcapsules. LCEO has the best inhibition effect on molds and the worst inhibition effect on yeasts. At 50 °C, essential oil and its microcapsules exhibit the least change in the inhibition diameter of the studied bacteria, which indicates an effective inhibitory action. A microcapsule release investigation revealed that LRCD was an appropriate wall material for controlling the staggered release of essential oil and extending the strain’s inhibitory period. The first-order kinetic equation is more consistent with higher temperatures during the release phase, whereas the Peppas equation is more consistent with lower temperatures. This was the first study on the LCEO/LRCD complex’s role in the development and suppression of bacteria and fungi.

## Figures and Tables

**Table 1:** Volatile compounds of LCEO in original and inclusion complexes.

LCEO			
Volatiles in LCEO (%)		Volatiles in Microcapsules	
Volatiles	Area %	Volatiles	Area %
α-Citral	20.17 ± 0.25	Limonene	21.56 ± 0.15
β-Citral isomer	17.08 ± 0.21	α-Citral	20.09 ± 0.18
Limonene	15.94 ± 0.23	β-Citral isomer	16.91 ± 0.21
β-pinene	5.83 ± 0.12	β-Pinene	6.15 ± 0.15
β-Phellandrene	4.34 ± 0.09	β-Phellandrene	4.51 ± 0.11
(1R)-(+)-α-Pinene	3.28 ± 0.02	(1R)-(+)-α-Pinene	3.82 ± 0.16
Eucalyptol	3.12 ± 0.05	Eucalyptol	2.75 ± 0.11
Carvone	2.94 ± 0.03	Linalool	2.63 ± 0.05
Carveol	2.83 ± 0.02	Sabinene	2.41 ± 0.04
1,8-Cineol	2.75 ± 0.05	Methyl heptenone	1.95 ± 0.25
Dihydrocarvone	2.54 ± 0.03	Citronellal	1.62 ± 0.05
E-β-Caryophyllene	2.45 ± 0.05	Carvone	1.23 ± 0.03
δ-3-Carene	2.22 ± 0.04	Carveol	1.01 ± 0.02
α-Cadinadien	1.83 ± 0.06	1,8-Cineol	0.94 ± 0.05
Linalool	1.35 ± 0.05	Dihydrocarvone	0.68 ± 0.03
Sabinene	1.34 ± 0.05	E-β-Caryophyllene	0.54 ± 0.04
Methyl heptenone	1.12 ± 0.02	Delta-3-Carene	0.49 ± 0.03
Citronellal	1.05 ± 0.01	α-Cadinadien	0.32 ± 0.02
4-Methyl-1,4-heptadiene	1.02 ± 0.02	Borneol	0.25 ± 0.05
Citronellal isomer	0.97 ± 0.02	Copaene	0.19 ± 0.05
1,5,9,11-Tridecatetraene,12-methyl-	0.82 ± 0.05	Menthone	0.17 ± 0.01
Terpineol	0.79 ± 0.05	Bicycloelemene	0.13 ± 0.02
Borneol	0.72 ± 0.03		
Copaene	0.65 ± 0.04		
Menthone	0.59 ± 0.06		
Bicycloelemene	0.53 ± 0.05		
Pelargonaldehyde	0.49 ± 0.05		
Azulene	0.34 ± 0.07		
β-Myrcene	0.32 ± 0.05		

Results are expressed as mean ± standard deviation of (*n* = 3) three independent experiments.

**Table 2:** Determination of the diameter of the inhibition zone of LCEO.

Test Strain	Diameter of Inhibition Zone in Direct Contact (mm)	Diameter of Inhibition Zone in Fumigation (mm)
*E. coli*	15.88 ± 0.05 ^b^	16.08 ± 0.28 ^a^
*B. subtilis*	15.51 ± 0.13 ^b^	18.33 ± 0.22 ^b^
*Kluyveromyces marxianus*	14.46 ± 0.14 ^a^	16.27 ± 0.15 ^a^
*A. niger*	20.53 ± 0.09 ^d^	23.28 ± 0.24 ^d^
*Rhizopusoryzae*	19.82 ± 0.16 ^c^	22.19 ± 0.09 ^c^

Results expressed as mean ± standard deviation (*n* = 3), with different superscript letters in the same column indicating significant differences (*p* < 0.05).

**Table 3:** Determination of the diameter of the inhibition zone of the LCEO and microcapsules at different temperatures.

Antimicrobial Agent	T (°C)	Diameter of the Inhibition Zone (mm)				
		*E. coli*	*B. subtilis*	*Kluyveromyces marxianus*	*A. niger*	*Rhizopus oryzae*
LCEO	4	15.01 ± 0.17 ^c^	13.13 ± 0.09 ^b^	13.81 ± 0.19 ^e^	22.13 ± 0.29 ^f^	20.06 ± 0.19 ^c^
	50	15.47 ± 0.13 ^cd^	13.89 ± 0.17 ^cd^	14.29 ± 0.08 ^f^	22.43 ± 0.18 ^f^	20.51 ± 0.23 ^cd^
	100	15.08 ± 0.09 ^c^	13.25 ± 0.12 ^b^	14.16 ± 0.18 ^f^	22.28 ± 0.07 ^f^	20.23 ± 0.13 ^c^
LCEO/LRCD	4	15.46 ± 0.13 ^cd^	13.62 ± 0.23 ^c^	15.12 ± 0.14 ^g^	22.21 ± 0.09 ^f^	20.89 ± 0.24 ^d^
	50	16.47 ± 0.21 ^e^	14.89 ± 0.18 ^f^	15.88 ± 0.14 ^h^	23.29 ± 0.18 ^g^	21.26 ± 0.17 ^e^
	100	16.24 ± 0.12 ^de^	14.56 ± 0.19 ^e^	15.63 ± 0.09 ^h^	22.45 ± 0.17 ^f^	20.81 ± 0.16 ^cd^

Results expressed as the mean ± standard deviation (*n* = 3), with different superscript letters in the same columns indicating significant differences (*p* < 0.05).

**Table 4:** Determination of the antimicrobial performance of LCEO and its microcapsules in direct contact.

Test Strain	LCEO		LCEO/LRCD	
	MIC mg/mL	MBC mg/mL	MIC mg/mL	MBC mg/mL
*E. coli*	3.0 ± 0.17 ^d^	6.0 ± 0.06 ^d^	6.0 ± 0.11 ^c^	12.0 ± 0.13 ^d^
*B. subtilis*	2.8 ± 0.07 ^c^	5.6 ± 0.12 ^c^	8.4 ± 0.12 ^e^	11.2 ± 0.09 ^d^
*Kluyveromyces marxianus*	3.5 ± 0.11 ^e^	7.0 ± 0.13 ^e^	7.0 ± 0.09 ^d^	7.0 ± 0.17 ^b^
*A. niger*	1.8 ± 0.03 ^a^	3.6 ± 0.04 ^a^	3.6 ± 0.02 ^a^	3.6 ± 0.07 ^a^
*Rhizopus oryzae*	2.3 ± 0.06 ^b^	4.6 ± 0.08 ^b^	4.6 ± 0.05 ^b^	9.2 ± 0.12 ^c^

Results expressed as mean ± standard deviation (*n* = 3), with different superscript letters in the same column indicating significant differences (*p* < 0.05).

**Table 5:** Kinetic release parameters of the inclusion complex at 37 and 65 °C.

Release Kinetic Equation	65 °C	37 °C
Zero-order dynamic equation: Q = kt	Q = 0.50t + 25.16 R^2^ = 0.75	Q = 0.49t + 12.43 R^2^ = 0.91
First-order dynamic equation: Q = 1 − etp^(−kt)^	Q = 58.01 (1 − etp^−2.48t^) R^2^ = 0.87	Q = 43.26 (1 − etp^−0.83t^) R^2^ = 0.80
Higuchi equation: Q = kt^0.5^	Q = 6.84t^0.5^ + 6.64 R^2^ = 0.94	Q = 6.25t^0.5^ − 2.51 R^2^ = 0.99
Peppas equation: Q = kt^n^	Q = 14.81t^0.35^ − 1.70 R^2^ = 0.96	Q = 5.33t^0.53^ − 1.27 R^2^ = 0.99

**Table 6:** Effect of slow-release time on antimicrobial performance.

Diameter of Antimicrobial Circle (mm)				
Sample	Day	*E. coli*	*A. niger*	*Rhizopus oryzae*
LCEO	3	9.25 ± 0.48 ^a^	8.64 ± 0.69 ^a^	12.87 ± 0.75 ^a^
	7	22.93 ± 1.12 ^d^	24.38 ± 2.18 ^d^	44.82 ± 0.54 ^e^
	14	31.93 ± 1.43 ^e^	65.16 ± 1.31 ^i^	>90
LCEO/LRCD Inclusion complex	3	10.11 ± 0.48 ^b^	13.57 ± 0.23 ^ab^	19.12 ± 0.34 ^b^
	7	22.65 ± 0.54 ^d^	28.87 ± 1.12 ^e^	41.34 ± 0.42 ^d^
	14	31.74 ± 0.62 ^e^	52.76 ± 1.18 ^e^	82.76 ± 1.08 ^g^
control group	3	12.32 ± 1.02 ^b^	18.33 ± 0.67 ^c^	58.64 ± 0.65 ^f^
	7	25.32 ± 0.76 ^d^	47.76 ± 0.54 ^g^	>90
	14	35.32 ± 0.23 ^f^	82.16 ± 0.15 ^j^	>90

Results expressed as mean ± standard deviation (*n* = 3), with different superscript letters in the same column indicating significant differences (*p* < 0.05).

## Data Availability

Data is contained within the article.

## Abbreviations

LRCD: large-ring vyclodextrins; LCEO, *Litsea cubeba* essential oil; FTIR, Fourier transform infrared spectroscopy; TGA, Thermogravimetric analysis; GC-MS, Gas chromatography–mass spectrometry.
